# MSCF-Net: A Vision Mamba Network with Multi-Scale Context Bridging and Cross-Layer Adaptive Fusion for Medical Image Segmentation

**DOI:** 10.3390/jimaging12070299

**Published:** 2026-07-03

**Authors:** Jiahao Guo, Tao Chen, Jiaxi Hu, Yuanhong Zhou

**Affiliations:** School of Mathematics and Computer Science, Shaanxi University of Technology, Hanzhong 723001, China; guojiahao@snut.edu.cn (J.G.); hujiaxi@snut.edu.cn (J.H.); zhouyuanhong@snut.edu.cn (Y.Z.)

**Keywords:** medical image segmentation, Vision Mamba, state space model, multi-scale context, adaptive fusion, skin lesion segmentation, polyp segmentation

## Abstract

Accurate medical image segmentation remains challenging when lesions have large-scale variation, weak boundaries, and strong background interference. Vision Mamba provides efficient long-range modeling, but current Mamba-based U-shaped networks are still limited by weak local multi-scale representation and coarse skip fusion. This study proposes MSCF-Net, a Vision Mamba segmentation network for dermoscopic and endoscopic images. The network is built on VM-UNet and introduces two modules. The Multi-Scale Context Bridging (MSCB) module enriches bottleneck features with local, dilated, and global context. The Cross-Layer Adaptive Fusion (CLAF) module recalibrates encoder–decoder features in channel and spatial dimensions, reducing noisy shallow feature transmission. A structure loss is used to improve region completeness and boundary quality. Experiments on ISIC 2017, ISIC 2018, and CVC-ClinicDB show Dice scores of 90.62%, 90.82%, and 91.72%, and mIoU values of 82.02%, 82.31%, and 84.56%, respectively. Compared with representative baselines evaluated in our experiments, MSCF-Net achieves competitive segmentation performance under the adopted benchmark protocol. Ablation, qualitative, and spatial response analyses further indicate that MSCB improves scale-aware representation, while CLAF helps the decoder focus on lesion-related cues. The results suggest that MSCF-Net provides a favorable accuracy–efficiency trade-off for medical image segmentation.

## 1. Introduction

Automated medical image segmentation is a core step in computer-aided diagnosis. In dermoscopic images, lesions often show fuzzy boundaries, low contrast, and hair occlusion. In endoscopic images, polyps are affected by folds, mucus, highlights, and irregular shape. In both settings, segmentation quality directly influences later diagnosis and treatment planning. Over the last decade, the field has moved from handcrafted features to deep learning. Among existing models, U-Net and its variants remain the dominant framework because they combine strong local modeling with effective encoder–decoder fusion [1].

Despite their success, convolutional networks are still limited by local receptive fields. Long-range dependencies are usually learned only after stacking many layers. This can reduce segmentation stability when lesion scale changes sharply or boundaries are unclear. Transformer-based models were therefore introduced to model global relationships through self-attention [2,3]. However, their quadratic complexity remains a practical burden for high-resolution dense prediction.

Recent state space models, especially Mamba, provide a better trade-off between global modeling and computational cost [4]. VM-UNet showed that a Vision Mamba backbone can work well for medical image segmentation [5]. Even so, current Mamba-based U-shaped networks still have two clear limitations. First, the bottleneck features remain largely single-scale, so they cannot describe both fine lesion edges and large lesion structures equally well. Second, skip connections are often fused by direct summation or concatenation. This makes it easy for shallow noise to enter the decoder.

To address these issues, we propose MSCF-Net, a Vision Mamba medical image segmentation network with Multi-Scale Context Bridging (MSCB) and Cross-Layer Adaptive Fusion (CLAF). MSCB enriches the bottleneck with complementary receptive fields. CLAF filters skip features through joint channel and spatial attention. We also adopt a structure loss to improve region completeness and boundary quality.

The main contributions of this work are summarized as follows:

- We propose a multi-scale contextual bridging strategy that compensates for the limited local-scale sensitivity of Vision Mamba bottleneck features.
- We design a cross-layer adaptive fusion module that recalibrates encoder–decoder features from both channel and spatial dimensions, reducing noisy shallow feature transmission.
- We validate MSCF-Net on dermoscopic and endoscopic segmentation benchmarks, showing that the proposed modules consistently improve VM-UNet with only a small increase in GFLOPs.

## 2. Related Work

### 2.1. CNN-Based Medical Image Segmentation

Early deep segmentation methods were largely driven by fully convolutional networks [6]. U-Net established the standard symmetric encoder–decoder design with skip connections and remains the basis of numerous medical segmentation systems [1]. Many subsequent improvements focused on stronger feature fusion, boundary recovery, or multi-scale context modeling. Representative examples include UNet++ [7], Attention U-Net [8], UNet 3+ [9], DeepLabV3+ [10], CE-Net [11], ResUNet++ [12], and nnU-Net [13]. For polyp segmentation, PraNet introduced parallel decoding and reverse attention to enhance region localization and boundary refinement [14]. These CNN-based models remain highly effective at capturing local texture and edge information, but they are less efficient at modeling long-range spatial dependencies.

### 2.2. Transformer-Based Medical Image Segmentation

Transformers were introduced to medical segmentation to compensate for the limited receptive field of CNNs. Vision Transformer provides a purely attention-based image representation [3], and hybrid architectures such as TransUNet combine convolutional local modeling with Transformer-based global reasoning [15]. Swin Transformer reduces attention cost by window partitioning [16], and later models such as TransFuse [17], UCTransNet [18], UNETR [19], and Swin UNETR [20] extended Transformer segmentation to richer cross-layer fusion and 3D medical scenarios. Although Transformers model long-range relationships effectively, they often require higher memory and computation, and their weak local inductive bias can harm precise boundary recovery in limited-data settings.

### 2.3. State Space Model-Based Medical Image Segmentation

State space models have recently emerged as an efficient alternative for long-range dependency modeling. Mamba introduces selective scanning while retaining linear complexity [4], and S4 provides an important structured state space foundation for this family of methods [21]. In medical image segmentation, U-Mamba and SegMamba combined Mamba-style modeling with medical segmentation backbones to improve global context perception [22,23]. VM-UNet and Mamba-UNet then showed that Vision Mamba-style U-shaped architectures can be effective for medical image segmentation [5,24]. Follow-up studies such as VM-UNetV2, LKM-UNet, Swin-UMamba, and H-VMUNet further explored semantic-detail infusion, locality enhancement, windowed scanning, and high-order selective scanning designs [25,26,27,28]. These recent studies show that Mamba-based segmentation is developing rapidly, but they also indicate that long-range modeling alone is not sufficient for difficult lesion segmentation. Local-scale variation at the bottleneck and noisy cross-layer feature transmission still need to be handled explicitly. Therefore, MSCF-Net focuses on combining Vision Mamba representation with multi-scale bottleneck compensation and adaptive skip fusion.

## 3. Materials and Methods

### 3.1. Preliminary: Vision State Space Modeling

Modern state space model architectures rely on a continuous linear dynamical system that maps an input sequence x(t) to an output y(t) through an implicit state h(t):

(1) $$\begin{array}{cc} \dot{h}\left(t\right) & =Ah(t)+Bx(t),\\ y(t) & =Ch(t), \end{array}$$

where *A* is the state matrix and *B* and *C* are projection parameters. To use this formulation in deep learning, the continuous system is discretized with a step size Δ. Using zero-order hold, the discrete transition can be written as

(2) $$\bar{A}=\exp\left(\Delta A\right),\;\bar{B}={\left(\Delta A\right)}^{-1}\left(\exp(\Delta A)-I\right)\Delta B.$$

After discretization, the model can be computed recurrently or through a structured convolution:

(3) $$\begin{array}{cc} h_{t} & =\bar{A}h_{t-1}+\bar{B}x_{t},\\ y_{t} & =Ch_{t}. \end{array}$$

The equivalent convolutional kernel is

(4) $$\bar{K}=\left(C\bar{B},C\bar{A}\bar{B},\ldots ,C{\bar{A}}^{L-1}\bar{B}\right),\;y=x*\bar{K},$$

where *L* is the sequence length and ∗ denotes convolution. This mechanism enables efficient long-range context aggregation while preserving linear complexity [4,21].

### 3.2. Overall Architecture

**Figure 1:**
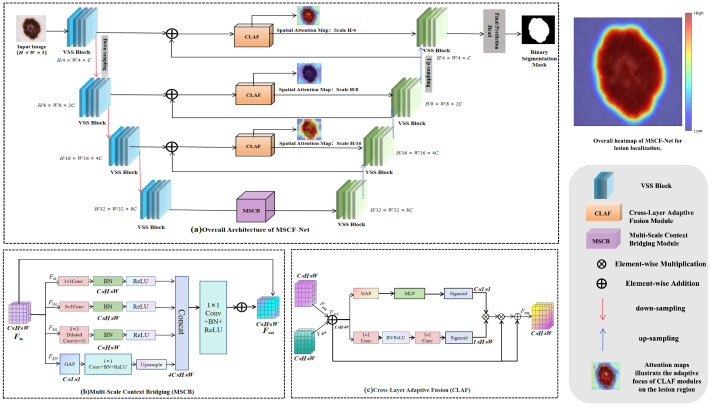
Overall architecture of MSCF-Net. Panel (**a**) shows the symmetric U-shaped encoder–decoder built with VSS blocks. Panel (**b**) presents the Multi-Scale Context Bridging module inserted at the bottleneck. Panel (**c**) shows the Cross-Layer Adaptive Fusion module used to replace naive skip connections.

MSCF-Net follows a symmetric U-shaped encoder–decoder architecture built around Visual State Space (VSS) blocks, as illustrated in Figure 1. To balance computational efficiency and representation depth, the encoder stages follow the depth configuration [2,2,2,2]. The input image is first projected by a patch embedding layer and then processed by a hierarchical encoder. At the bottleneck, we insert the proposed MSCB module to enrich deep features with complementary receptive fields. During decoding, patch expansion progressively restores spatial resolution. Instead of using a naive skip connection, we introduce CLAF to adaptively fuse shallow encoder features with deep decoder features, thereby improving lesion-focused reconstruction.

### 3.3. VSS Block as the Backbone Unit

The VSS block serves as the basic feature extractor in both the encoder and the decoder. By means of directional selective scanning, the block captures long-range spatial context under linear complexity, which gives MSCF-Net strong global modeling capacity. In our design, VSS blocks are responsible for the backbone representation learning, while MSCB and CLAF specifically address the local multi-scale and cross-layer fusion limitations that remain in complex medical images.

### 3.4. Multi-Scale Context Bridging Module

Medical lesions show strong scale variability, and single-receptive-field operators are often unable to represent both microscopic edge details and macroscopic object structure. This problem is especially severe at the bottleneck, where repeated downsampling compresses spatial information. We therefore introduce the Multi-Scale Context Bridging (MSCB) module to inject explicit multi-scale inductive bias into the bottleneck representation.

MSCB contains four parallel branches. The identity branch preserves the original semantic stream by a 1×1 convolution. The local branch uses a standard 3×3 convolution to capture fine-grained texture. The dilated branch adopts a 3×3 atrous convolution with dilation rate 3 to expand the receptive field without heavy overhead, following the motivation of multi-scale context aggregation [10]. The global branch uses global average pooling followed by a 1×1 projection and upsampling to inject image-level prior information. The four branches are defined as

(5) $$\left\{\begin{array}{cc} F_{\mathrm{id}} & =\delta \;\left(\mathrm{BN}\;\left({\mathrm{Conv}}_{1\times 1}\left(F_{\mathrm{in}}\right)\right)\right),\\ F_{\mathrm{loc}} & =\delta \;\left(\mathrm{BN}\;\left({\mathrm{Conv}}_{3\times 3}\left(F_{\mathrm{in}}\right)\right)\right),\\ F_{\mathrm{dil}} & =\delta \;\left(\mathrm{BN}\;\left({\mathrm{DConv}}_{3\times 3,r=3}\left(F_{\mathrm{in}}\right)\right)\right),\\ F_{\mathrm{glo}} & =\mathrm{Up}\;\left(\delta \;\left(\mathrm{BN}\;\left({\mathrm{Conv}}_{1\times 1}\left(\mathrm{GAP}\left(F_{\mathrm{in}}\right)\right)\right)\right)\right), \end{array}\right.$$

where δ denotes the ReLU activation and BN denotes batch normalization. As shown in Equation (5), the identity, local, dilated, and global branches all use BN followed by ReLU after their convolutional projection. Therefore, $F_{\mathrm{loc}}$ and $F_{\mathrm{dil}}$ differ from $F_{\mathrm{id}}$ in kernel size and receptive field rather than in the activation function: $F_{\mathrm{id}}$ uses a 1×1 projection, $F_{\mathrm{loc}}$ uses a standard 3×3 convolution, and $F_{\mathrm{dil}}$ uses a 3×3 dilated convolution with rate 3. The branch outputs are concatenated and fused through a 1×1 convolution, and a residual connection is added:

(6) $$F_{\mathrm{out}}=\delta \;\left(\mathrm{BN}\;\left({\mathrm{Conv}}_{1\times 1}\;\left([F_{\mathrm{id}},F_{\mathrm{loc}},F_{\mathrm{dil}},F_{\mathrm{glo}}]\right)\right)\right)+F_{\mathrm{in}}.$$

This design forms a complete feature chain from local pixel-level details to global image-level context, which helps the network handle lesions of different sizes. Unlike generic ASPP-style context modules, MSCB is placed specifically at the Vision Mamba bottleneck. Its role is not only to enlarge the receptive field, but also to compensate for the loss of local scale cues after repeated downsampling and selective scanning.

### 3.5. Cross-Layer Adaptive Fusion Module

To bridge the semantic gap between encoder and decoder features while suppressing shallow background noise, we propose the Cross-Layer Adaptive Fusion (CLAF) module. CLAF first initializes a fused feature by element-wise addition:

(7) $$F_{\mathrm{init}}=F_{\mathrm{enc}}+F_{\mathrm{dec}}.$$

It then computes channel attention $M_{c}$ and spatial attention $M_{s}$ in parallel. Channel attention compresses the spatial dimensions through global average pooling and uses a two-layer 1×1 MLP to model inter-channel dependency, which is conceptually related to SE-style feature recalibration [29]. Spatial attention uses a bottleneck projection to estimate the importance of each spatial position, similar in spirit to convolutional block attention [30]. The two weights are formulated as

(8) $$\left\{\begin{array}{cc} M_{c} & =\sigma \;\left({\mathrm{Conv}}_{1\times 1}\;\left(\delta \;\left({\mathrm{Conv}}_{1\times 1}\left(\mathrm{GAP}\left(F_{\mathrm{init}}\right)\right)\right)\right)\right),\\ M_{s} & =\sigma \;\left({\mathrm{Conv}}_{1\times 1}\;\left(\delta \;\left(\mathrm{BN}\;\left({\mathrm{Conv}}_{1\times 1}\left(F_{\mathrm{init}}\right)\right)\right)\right)\right), \end{array}\right.$$

where σ is the Sigmoid function. The final output is obtained by multiplicative reweighting plus a residual connection:

(9) $$F_{\mathrm{out}}=F_{\mathrm{init}}⊗\left(M_{c}⊗M_{s}\right)+F_{\mathrm{init}},$$

where ⊗ denotes element-wise multiplication. In this way, CLAF turns passive skip fusion into active feature selection and improves decoder purity in the presence of hair, shadow, mucus, or specular noise. CLAF also differs from standard SE or CBAM modules. SE and CBAM usually recalibrate a single feature map, whereas CLAF operates on cross-layer encoder–decoder features. Its attention weights are therefore used for both feature enhancement and suppression of noisy shallow skip information before decoder reconstruction.

Overall, the novelty of MSCF-Net lies not in adding a generic attention or context block, but in identifying and addressing two specific weaknesses of Vision Mamba U-shaped segmentation: bottleneck scale compression and noisy skip transmission. MSCB and CLAF are designed around these two weaknesses and are therefore coupled to the Vision Mamba encoder–decoder structure. In other words, MSCB is used as a bottleneck compensation module for scale-compressed Vision Mamba features, while CLAF is used as a cross-layer filtering module for encoder–decoder feature transmission. This design differs from applying standalone multi-scale or attention modules to a single feature map.

### 3.6. Structure Loss

To better handle irregular lesion boundaries and class imbalance, we optimize MSCF-Net with the structure loss adopted from PraNet [14]. The final loss combines binary cross-entropy and IoU terms:

(10) $$L_{\mathrm{str}}=L_{\mathrm{BCE}}+L_{\mathrm{IoU}}.$$

Given a prediction probability map *P* and a ground-truth mask *G*, the BCE term is

(11) $$L_{\mathrm{BCE}}=-\frac{1}{N}\sum_{i,j}\left[G_{i,j}\log\left(P_{i,j}\right)+\left(1-G_{i,j}\right)\log\left(1-P_{i,j}\right)\right],$$

and the IoU term is

(12) $$L_{\mathrm{IoU}}=1-\frac{\sum_{i,j}P_{i,j}G_{i,j}+\epsilon }{\sum_{i,j}P_{i,j}+\sum_{i,j}G_{i,j}-\sum_{i,j}P_{i,j}G_{i,j}+\epsilon },$$

where ϵ is a smoothing constant. Because the model output is already passed through Sigmoid, the loss is computed directly on probabilities to avoid redundant activation.

## 4. Experiments

### 4.1. Datasets and Evaluation Metrics

We evaluate MSCF-Net on three public medical image segmentation benchmarks: ISIC 2017 [31], ISIC 2018 [32], and CVC-ClinicDB [33]. ISIC 2017 and ISIC 2018 are dermoscopic lesion segmentation datasets characterized by fuzzy boundaries, strong scale changes, low local contrast, and frequent hair occlusion. CVC-ClinicDB is a polyp segmentation dataset with more complex background texture, stronger illumination artifacts, and small irregular targets. Together, these datasets provide a useful test bed for evaluating performance across different benchmark settings.

For data partitioning, ISIC 2017 contains 2150 dermoscopic images with segmentation masks, including 1500 training images and 650 testing images. ISIC 2018 contains 2694 dermoscopic images with segmentation masks, including 1886 training images and 808 testing images. For these two datasets, we followed the data partition protocol used in VM-UNet to ensure a direct comparison with the most closely related Vision Mamba baseline. CVC-ClinicDB contains 612 annotated colonoscopy polyp images and was split at the image level with a fixed 8:2 ratio, yielding 489 training images and 123 testing images. The same splits were used for MSCF-Net and all reproduced baselines. Because complete patient-level or sequence-level identifiers are not available in the processed benchmark split, potential frame-level or near-duplicate overlap cannot be fully excluded from the available metadata.

We report five evaluation metrics: mean intersection over union (mIoU), Dice similarity coefficient (DSC), pixel accuracy (Acc), specificity (Spe), and sensitivity (Sen). mIoU and DSC measure the overlap between prediction and ground truth, whereas Acc, Spe, and Sen characterize overall classification correctness, background suppression, and lesion recall ability, respectively.

### 4.2. Implementation Details

All experiments were conducted on a workstation equipped with an NVIDIA GeForce RTX 4090 GPU (Santa Clara, CA, USA) and implemented using PyTorch 1.13.0 with CUDA 11.7 [34]. Images were resized to 256×256 during both training and inference. Online augmentation included random flipping, rotation, and scaling to improve the tolerance to lesion shape variation and imaging disturbance. For fair comparison, all compared methods in Table 1, Table 2 and Table 3 were evaluated under the same preprocessing, image size, augmentation strategy, optimizer, training epochs, and data partitions. No results in Table 1, Table 2 and Table 3 were directly copied from the original papers; all reported scores were obtained from our own experiments under the unified evaluation protocol. The baseline architectures were implemented and trained according to their original model settings while keeping the evaluation protocol consistent across methods. We trained all models for 300 epochs with a batch size of 32 under a fixed training protocol. The checkpoint from the final training epoch was used for final evaluation. No validation- or test-set-based checkpoint selection was performed, and the same checkpoint rule was applied to MSCF-Net and all reproduced baselines. No additional validation set was constructed beyond the adopted benchmark splits, and no validation-based hyperparameter search was performed after the training protocol was fixed. AdamW was used as the optimizer [35], and the learning rate was updated with cosine annealing [36]. The initial learning rate was set to $1\times 10^{-3}$ for ISIC 2017 and ISIC 2018, and $1\times 10^{-4}$ for CVC-ClinicDB. The structure loss in Equations (10)–(12) was used for all training runs. For the focused Mamba-family comparison, five repeated runs with different random seeds were conducted under the same protocol, and the results are reported as mean ± standard deviation. Parameter counts were computed from model parameters, and GFLOPs were measured using THOP with a single input tensor of size 1×3×256×256.

### 4.3. Comparison with State-of-the-Art Methods

We compare MSCF-Net with representative medical image segmentation models, including Att-U-Net [8], DeepLabV3+ [10], MA-Net [37], U-Net [1], UNet++ [7], TransUNet [15], VM-UNet [5], and EGE-UNet [38]. The TransUNet baseline was implemented according to its original model setting and trained under the same protocol as the other compared methods. As a representative Transformer-based medical image segmentation model, TransUNet combines Transformer-based global context modeling with a U-shaped decoding structure. Quantitative results on the three datasets are reported in Table 1, Table 2 and Table 3. MA-Net is omitted on CVC-ClinicDB because no comparable result under our reproduced setting was available.

On ISIC 2017, MSCF-Net achieves the best mIoU, DSC, Acc, and Sen, while maintaining very high specificity. MA-Net obtains the highest specificity on this dataset, which indicates a stronger tendency to classify background pixels conservatively. However, medical lesion segmentation usually requires a balanced overlap quality and lesion recall. The higher DSC and Sen of MSCF-Net show that it recovers lesion regions more completely while keeping background discrimination competitive. A similar trend appears on ISIC 2018, where MSCF-Net again obtains the best mIoU, DSC, and Acc. On CVC-ClinicDB, the proposed model reaches 84.56% mIoU and 91.72% DSC, showing competitive performance on the endoscopic benchmark.

**Figure 2:**
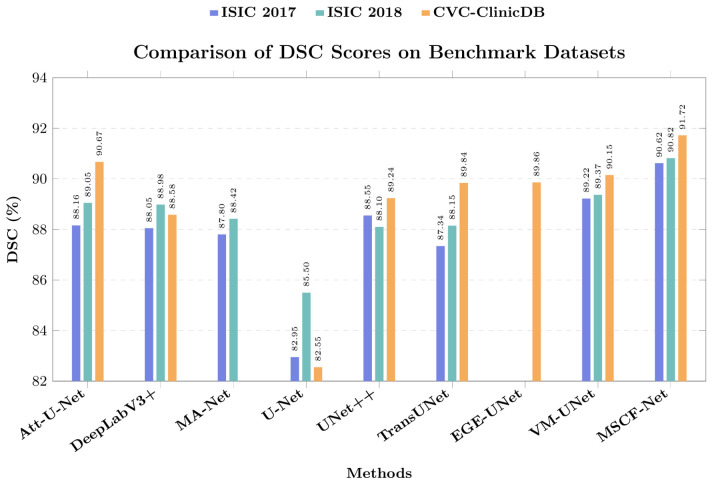
DSC comparison across three benchmark datasets. Higher values indicate better agreement between predictions and ground truth. Blank positions denote methods without comparable reproduced results on the corresponding dataset.

To visualize overall DSC behavior across datasets, Figure 2 summarizes the DSC values reported in Table 1, Table 2 and Table 3. MSCF-Net achieves the highest DSC among the compared methods in this benchmark comparison, which indicates competitive segmentation quality across the evaluated dermoscopic and endoscopic datasets. Blank positions indicate methods without comparable reproduced results on that dataset.

Among the compared methods, VM-UNet is the most directly related Mamba-based baseline, because MSCF-Net is built on the same Vision Mamba U-shaped framework. Compared with VM-UNet, MSCF-Net improves DSC by 1.40%, 1.45%, and 1.57% on ISIC 2017, ISIC 2018, and CVC-ClinicDB, respectively. These gains indicate that the proposed MSCB and CLAF modules improve the baseline not merely by increasing model size, but by enhancing multi-scale bottleneck representation and adaptive skip fusion.

To further respond to recent Mamba-based medical segmentation studies, we also provide a focused comparison with representative Mamba-family baselines in Table 4, including H-VMUNet [28], Mamba-UNet [24], VM-UNet [5], and VM-UNetV2 [25]. The table reports mIoU, DSC, Acc, Spe, and Sen as mean ± standard deviation over five repeated runs under the same benchmark protocol. MSCF-Net achieves the highest average mIoU and DSC on all three datasets and provides a balanced sensitivity–specificity profile. These results further support the effectiveness of adding MSCB and CLAF to the Vision Mamba encoder–decoder framework.

### 4.4. Complexity Analysis

Segmentation accuracy must be considered together with efficiency. Table 5 compares the parameter count, GFLOPs, and inference speed of different methods for an input size of 256×256. Parameter counts were computed from model parameters, and GFLOPs were measured using THOP with a single input tensor of size 1×3×256×256. FPS was measured on an NVIDIA GeForce RTX 4090 with batch size 1 after 50 warm-up iterations and 300 timed iterations repeated three times. MSCF-Net has 31.55 M parameters and 4.33 GFLOPs. Although its parameter count is higher than that of VM-UNet, its computational cost increases only slightly from 4.11 G to 4.33 G and remains lower than the reproduced CNN- and Transformer-based baselines. Compared with the newly included Mamba-family baselines, MSCF-Net keeps a moderate computational scale while achieving higher segmentation overlap on the evaluated benchmarks. MSCF-Net keeps a relatively low GFLOP level compared with the reproduced CNN- and Transformer-based baselines, but its measured FPS is not the highest among the compared methods. This suggests that the efficiency advantage of MSCF-Net is mainly reflected in computational complexity rather than raw inference throughput. The lower FPS may be related to the implementation overhead of Vision Mamba-style operations and the additional feature-fusion modules. Further implementation optimization will be considered in future work.

### 4.5. Ablation Study

The ablation study focuses on the contributions of the proposed MSCB and CLAF modules, their placement, and the computational cost of different module variants. All ablation results were obtained using the same fixed data partitions, unified training protocol, and final-epoch checkpoint rule. Table 6 shows that each module alone improves the baseline VM-UNet, and the joint model achieves the best DSC on all three datasets. This suggests that multi-scale bottleneck enrichment and adaptive skip fusion are complementary rather than redundant.

Next, we study module placement and configuration. Table 7 analyzes MSCB placement and CLAF scope under the full model setting. It shows that placing MSCB at the bottleneck is more effective than using it only at shallow stages, and that applying CLAF to all skip connections outperforms a single-skip version. The last row corresponds to the complete MSCF-Net configuration, where MSCB is placed at the bottleneck and CLAF is applied to all skip connections. The results support the final design choice used in MSCF-Net.

Finally, Table 8 compares the complexity and CVC-ClinicDB performance of the ablated variants. MSCB introduces the larger share of the extra parameters and computation, whereas CLAF adds lighter overhead. The combined model nevertheless offers the best accuracy-to-cost trade-off among the tested configurations.

### 4.6. Qualitative Analysis

**Figure 3:**
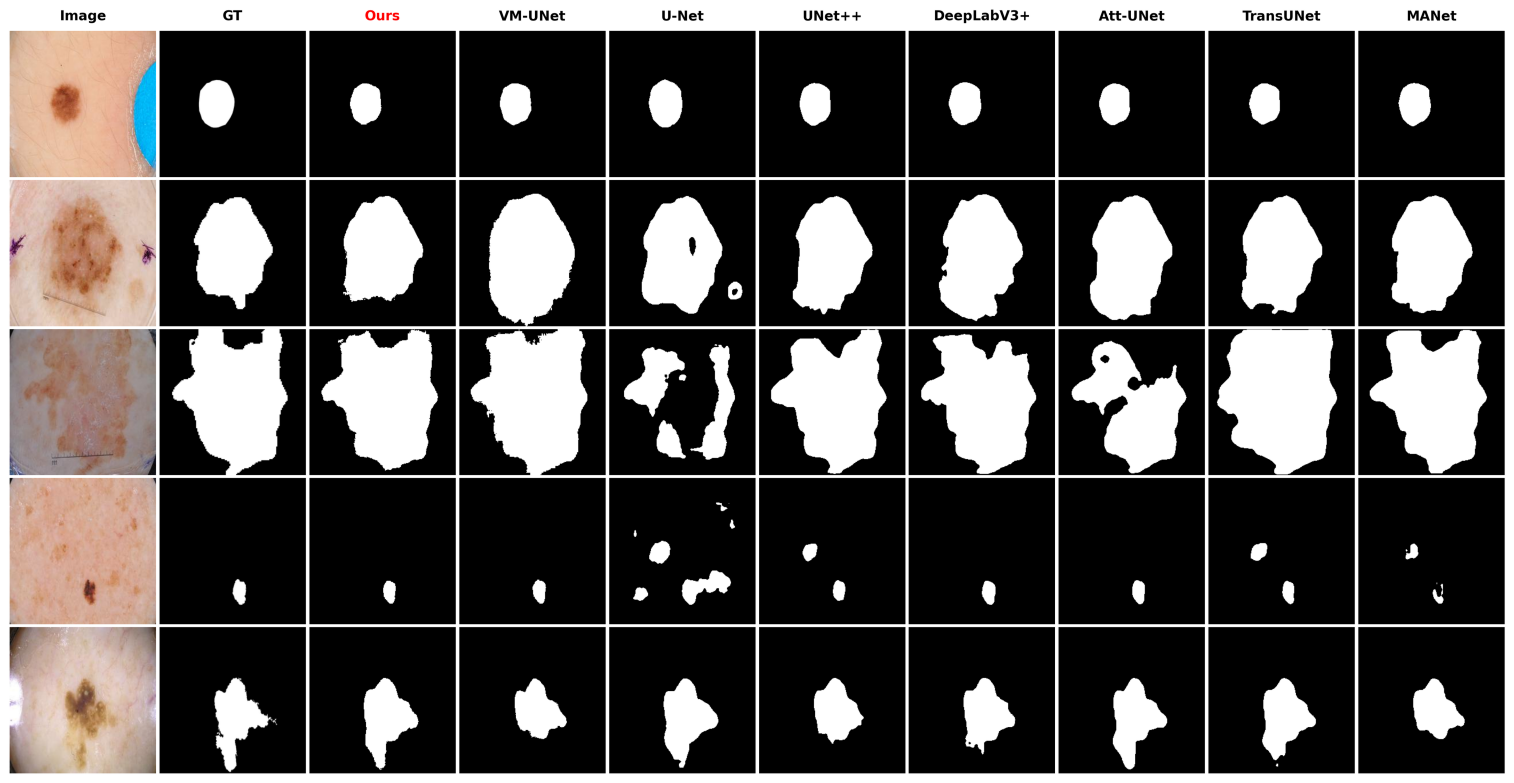
Qualitative comparison on ISIC 2017. “GT” denotes the ground-truth mask and “Ours” denotes MSCF-Net.

**Figure 4:**
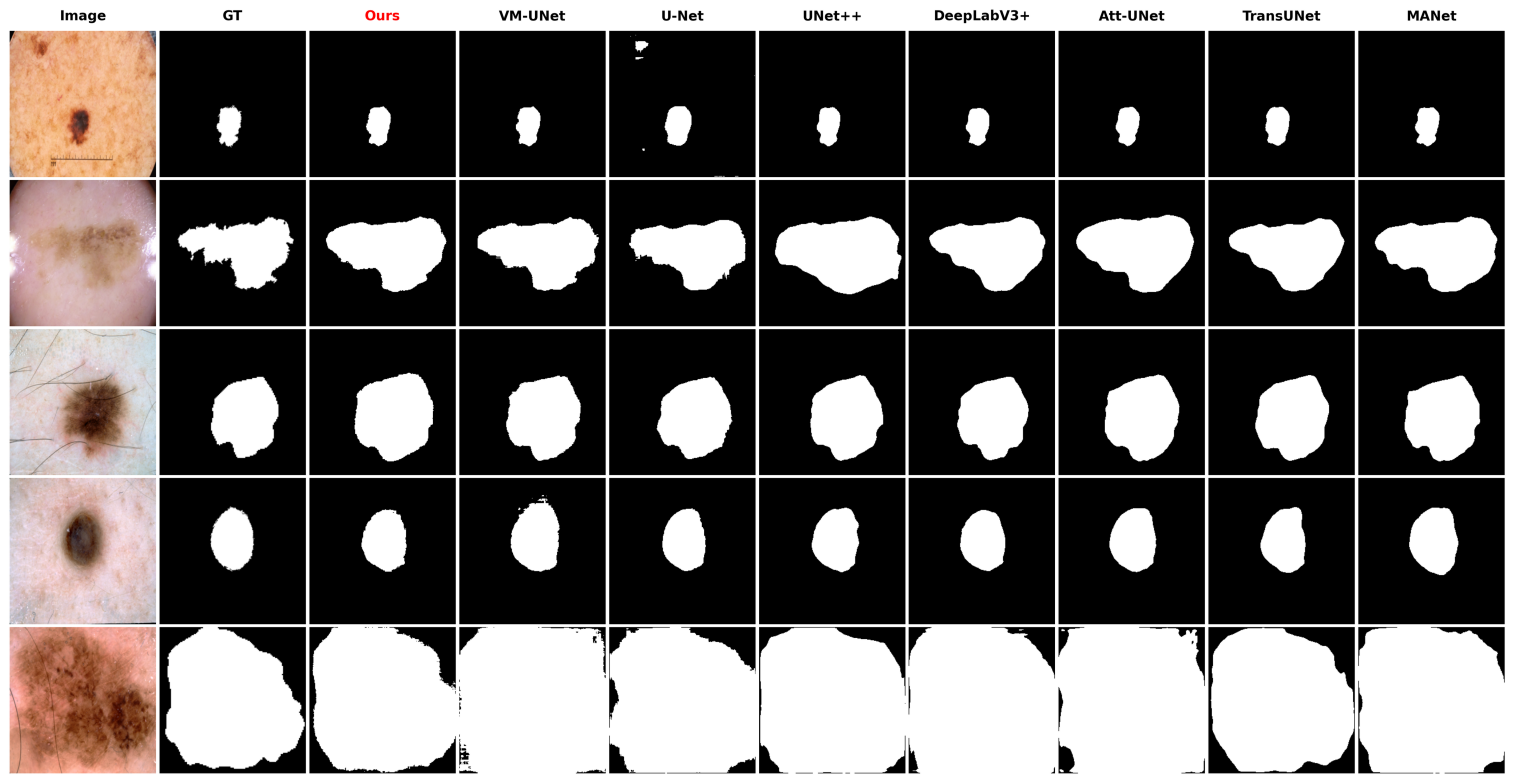
Qualitative comparison on ISIC 2018. MSCF-Net produces masks that remain more complete under larger appearance variation.

**Figure 5:**
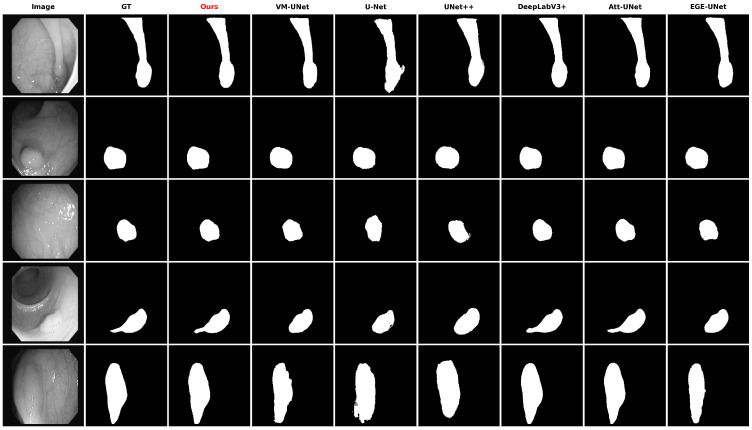
Qualitative comparison on CVC-ClinicDB. MSCF-Net shows better tolerance to highlights, irregular boundaries, and small target structures.

Figure 3, Figure 4 and Figure 5 compare visual predictions on dermoscopic and endoscopic samples. On ISIC 2017, the most obvious differences appear on small lesions and low-contrast boundaries, where several baselines either under-segment the lesion or produce fragmented masks. On ISIC 2018, complex appearance changes, color ambiguity, and larger lesion shape diversity make the task even harder, but MSCF-Net still recovers smoother and more complete lesion regions. On CVC-ClinicDB, the proposed model performs well on small polyps and samples with strong reflective artifacts. These qualitative observations suggest that CLAF may help reduce the influence of irrelevant shallow features, while MSCB may improve scale-aware representation.

We also observe several challenging and failure-prone situations. When a lesion is extremely small, very low-contrast, or has a boundary color that is close to the surrounding tissue, MSCF-Net may still produce slight under-segmentation. In endoscopic images, strong highlights and mucus-like structures can occasionally lead to local boundary expansion. These cases show that the model improves overall lesion completeness but does not fully solve all difficult boundary and appearance ambiguities.

**Figure 6:**
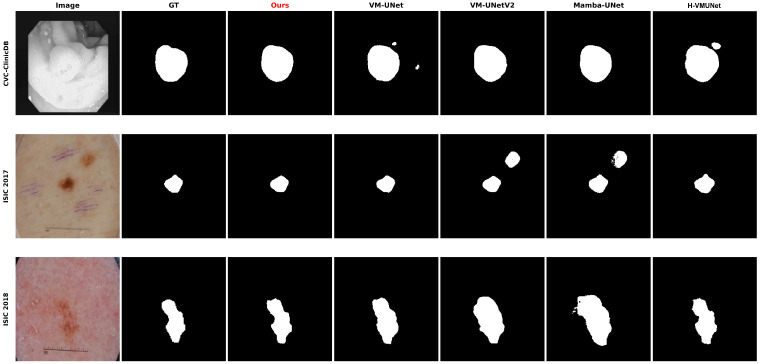
Qualitative comparison with recent Mamba-family segmentation baselines on CVC-ClinicDB, ISIC 2017, and ISIC 2018. “Ours” denotes MSCF-Net.

To further examine the behavior of recent Mamba-family segmentation baselines, Figure 6 compares MSCF-Net with VM-UNet, VM-UNetV2, Mamba-UNet, and H-VMUNet on representative samples from the three datasets. The examples show that MSCF-Net tends to preserve more complete target regions, while some Mamba-family baselines may produce fragmented masks, local over-segmentation, or missed lesion boundaries under low contrast, hair interference, and reflective endoscopic backgrounds.

**Figure 7:**
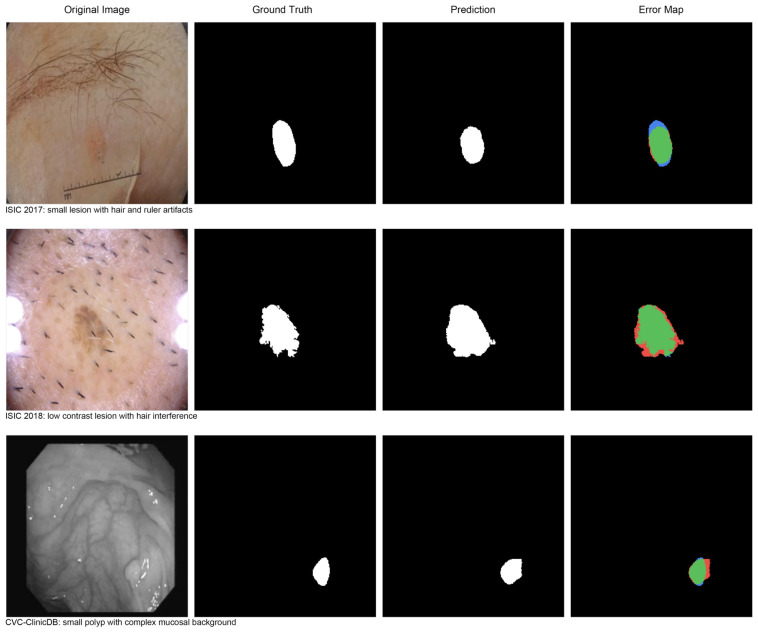
Challenging and failure-prone cases of MSCF-Net. In the error maps, green denotes correctly predicted foreground (true positive), red denotes false-positive regions, and blue denotes false-negative regions. These examples show remaining difficulties under small targets, low contrast, hair interference, and complex endoscopic backgrounds.

Figure 7 presents challenging and failure-prone cases of MSCF-Net. The green regions in the error maps denote correctly predicted foreground regions, while red and blue regions indicate local false-positive and false-negative areas. These examples suggest that very small lesions, low-contrast lesion boundaries, dense hair interference, and reflective or mucus-like endoscopic structures remain difficult. This observation is consistent with the limitations discussed below and indicates that external validation and more difficult clinical cases should be further studied.

### 4.7. Spatial Response and Probability-Map Visualization

To further analyze the spatial response of MSCF-Net, we visualized prediction-based lesion localization heatmaps generated from the final probability maps. Unlike gradient-based attribution methods such as Grad-CAM [39], this visualization focuses on the spatial distribution of the model’s final lesion confidence. Specifically, the predicted probability map was first normalized and binarized using an adaptive threshold. Morphological closing and opening operations were then applied to fill small holes and remove isolated noise. The largest connected component was retained as a coarse lesion prior and further smoothed by Gaussian filtering. Finally, the smoothed lesion prior was fused with the original probability map to obtain an overall localization heatmap, which was overlaid on the input image for visualization.

**Figure 8:**
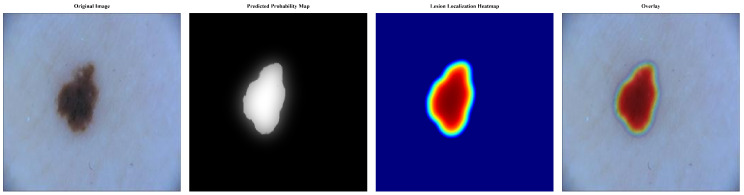
Prediction-based lesion localization visualization of MSCF-Net. The heatmaps are generated from the final predicted probability maps using adaptive thresholding, morphological refinement, connected-component selection, and Gaussian smoothing. Blue denotes low response, whereas yellow and red denote progressively higher lesion confidence.

As shown in Figure 8, the high-response regions are mainly concentrated inside the lesion areas, while the background regions receive much lower responses. This indicates that MSCF-Net can produce spatially coherent lesion predictions and maintain concentrated confidence responses around the main target regions under complex dermoscopic backgrounds. Although this visualization is not a gradient-based attribution analysis, it provides an intuitive view of the spatial confidence distribution produced by MSCF-Net.

**Figure 9:**
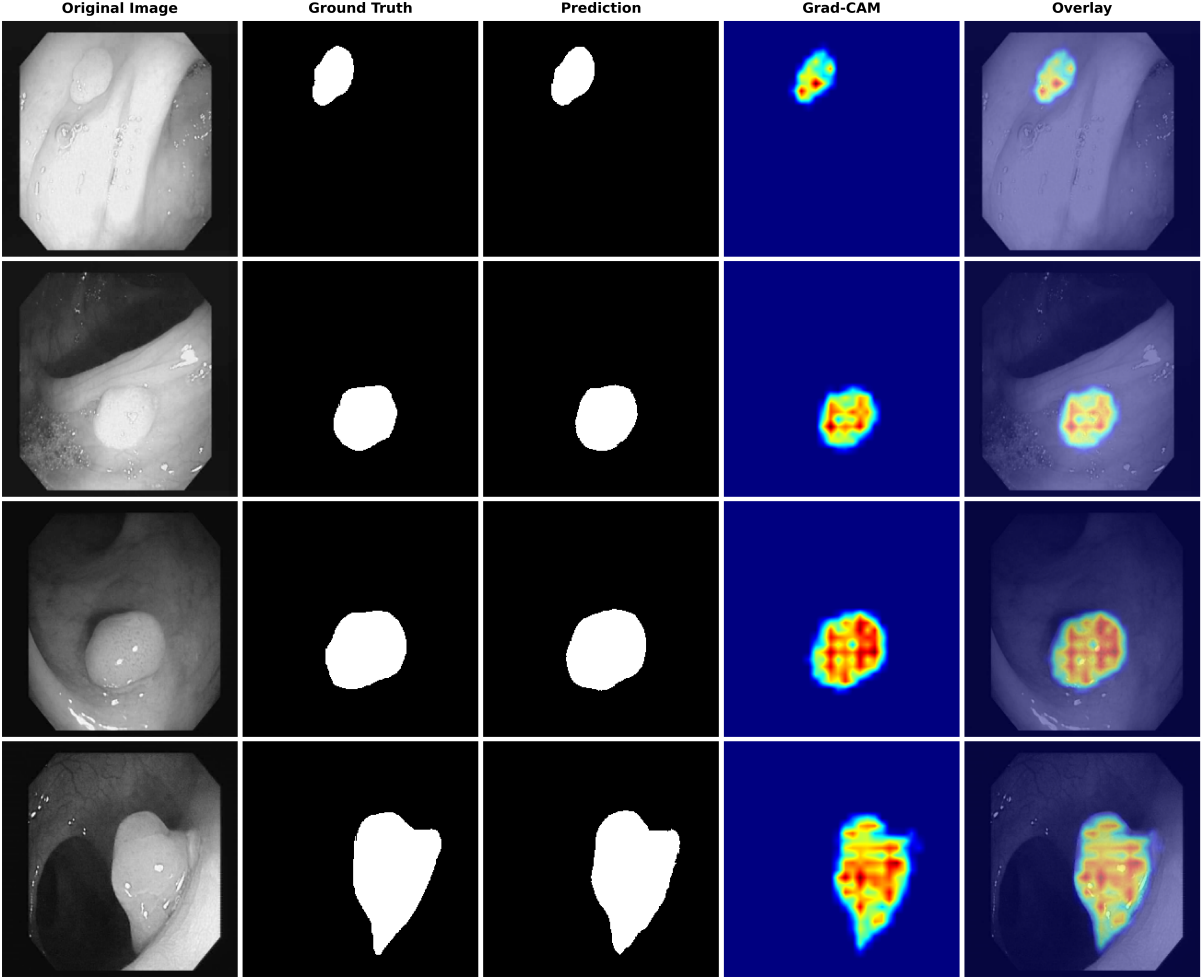
Grad-CAM visualization of MSCF-Net on representative CVC-ClinicDB samples. Blue denotes low activation, whereas yellow and red denote progressively higher activation. The heatmaps and overlays indicate that high-response regions are mainly concentrated around polyp structures.

As a complementary gradient-based visualization, we also used Grad-CAM to inspect whether the learned responses are concentrated around the target regions. Unlike the probability-map visualization in Figure 8, Grad-CAM is computed from internal model responses and is used here only as an auxiliary qualitative explanation. To keep the two visualization types distinct, Figure 8 shows the probability-map visualization on one representative example, whereas Figure 9 presents several CVC-ClinicDB examples to examine whether the response pattern is consistent across samples. As shown in Figure 9, the high-response areas are mainly located around the polyp regions, which provides additional visual evidence that MSCF-Net focuses on lesion-related structures rather than broad background areas.

### 4.8. Discussion

Across all experiments, MSCF-Net maintains a favorable balance between accuracy and efficiency. The quantitative gains are especially clear in DSC and sensitivity, which indicates that the proposed design improves both overlap quality and lesion recall. On the ISIC datasets, the multi-scale bottleneck is useful for recovering complete lesion regions under large appearance variation. On CVC-ClinicDB, the gain is also evident because endoscopic images often contain highlights, mucus, folds, and strong background texture. In this case, CLAF helps reduce noisy shallow skip information before decoder reconstruction.

The ablation results show that MSCB and CLAF contribute in different but complementary ways. MSCB mainly strengthens multi-scale semantic modeling at the bottleneck. CLAF improves decoder purity by filtering shallow cues that are not related to the target. Compared with the directly related VM-UNet baseline, these improvements show that explicit local-scale compensation and selective cross-layer fusion are both important for accurate lesion segmentation under the evaluated benchmark settings.

Although MSCF-Net does not always achieve the highest specificity, it provides a better balance between sensitivity and DSC, which is important for preserving lesion completeness in medical image segmentation. For example, MA-Net obtains the highest specificity on ISIC 2017, and Att-U-Net gives a slightly higher specificity on CVC-ClinicDB. However, the proposed method achieves stronger overlap quality and lesion recall, which are important for complete lesion-region segmentation. Since no clinical validation or reader study was conducted, the clinical usefulness of this balance still requires further evaluation.

Several limitations remain. No additional independent validation set was constructed beyond the adopted benchmark splits, and the reported results should therefore be interpreted under this fixed evaluation protocol. Extremely low-contrast lesions, targets with colors very close to the surrounding tissue, and very small objects may still be difficult. For CVC-ClinicDB, complete patient-level or sequence-level identifiers are unavailable in the processed split, so potential frame-level or near-duplicate overlap cannot be fully excluded from the available metadata. Boundary-level metrics such as HD95 and ASSD were not included in the current evaluation, and they will be considered in future work to provide a more detailed analysis of contour accuracy. The spatial response visualization is qualitative, so it should be supported by more quantitative explanation analysis in future studies. In addition, all experiments are based on public datasets, and public-dataset bias may limit the representativeness of real clinical acquisition conditions. External validation on multi-center dermoscopic and endoscopic cohorts is needed before clinical deployment.

## 5. Conclusions and Future Work

This work presented MSCF-Net, a Vision Mamba medical image segmentation network designed for lesions with scale variation, blurred boundaries, and strong background interference. The model combines MSCB at the bottleneck with CLAF across skip connections. In this way, it strengthens both multi-scale representation and adaptive cross-layer fusion. Experiments on ISIC 2017, ISIC 2018, and CVC-ClinicDB showed that MSCF-Net achieves competitive performance against VM-UNet and several strong CNN- and Transformer-based baselines in mIoU, DSC, and sensitivity under the adopted benchmark protocol.

Several directions remain for future work. First, deployment-oriented compression can be explored for real-time clinical systems. Second, broader validation on multi-center dermoscopic and endoscopic data is needed to evaluate performance consistency under real acquisition variability. Third, extending the design to 3D CT or MRI segmentation is a meaningful next step. Finally, prompt-guided segmentation and richer model explanation tools may further improve clinical usability and model transparency.

## Figures and Tables

**Table 1:** Quantitative comparison on ISIC 2017. An upward arrow (↑) indicates that a higher value is better. The best result in each column is shown in bold.

Model	mIoU (%) ↑	DSC (%) ↑	Acc (%) ↑	Spe (%) ↑	Sen (%) ↑
Att-U-Net	78.83	88.16	95.75	98.14	86.76
DeepLabV3+	78.65	88.05	95.53	97.23	89.13
MA-Net	78.25	87.80	95.61	**98.25**	85.68
U-Net	70.86	82.95	93.73	97.15	80.88
UNet++	79.45	88.55	95.79	97.18	90.58
TransUNet	77.53	87.34	95.32	97.46	87.26
VM-UNet	80.54	89.22	96.03	98.11	88.21
MSCF-Net (Ours)	**82.02**	**90.62**	**96.81**	98.14	**91.83**

**Table 2:** Quantitative comparison on ISIC 2018. An upward arrow (↑) indicates that a higher value is better. The best result in each column is shown in bold.

Model	mIoU (%) ↑	DSC (%) ↑	Acc (%) ↑	Spe (%) ↑	Sen (%) ↑
Att-U-Net	80.26	89.05	94.51	95.34	**91.89**
DeepLabV3+	80.15	88.98	94.59	96.08	89.89
MA-Net	79.25	88.42	94.27	95.59	90.10
U-Net	74.68	85.50	93.00	95.44	85.26
UNet++	78.73	88.10	94.09	95.33	90.16
TransUNet	78.82	88.15	94.60	**97.10**	86.67
VM-UNet	80.78	89.37	94.75	96.06	90.60
MSCF-Net (Ours)	**82.31**	**90.82**	**95.42**	96.55	91.83

**Table 3:** Quantitative comparison on CVC-ClinicDB. An upward arrow (↑) indicates that a higher value is better. The best result in each column is shown in bold.

Model	mIoU (%) ↑	DSC (%) ↑	Acc (%) ↑	Spe (%) ↑	Sen (%) ↑
Att-U-Net	82.93	90.67	98.13	**99.65**	85.87
DeepLabV3+	79.50	88.58	97.45	99.27	82.75
U-Net	70.28	82.55	97.07	99.58	76.82
UNet++	80.56	89.24	97.62	99.39	83.31
TransUNet	81.56	89.84	97.71	99.38	84.21
EGE-UNet	81.58	89.86	97.74	99.30	85.10
VM-UNet	82.07	90.15	97.85	99.16	87.28
MSCF-Net (Ours)	**84.56**	**91.72**	**98.43**	99.42	**90.45**

**Table 4:** Comparison with recent Mamba-based segmentation baselines. Results are reported as mean ± standard deviation (%) over five repeated runs. An upward arrow (↑) indicates that a higher value is better. The best result in each column is shown in bold.

CVC-ClinicDB					
**Model**	**mIoU** ↑	**DSC** ↑	**Acc** ↑	**Spe** ↑	**Sen** ↑
H-VMUNet	82.18±0.34	90.22±0.39	97.88±0.28	99.54±0.24	87.60±0.35
Mamba-UNet	83.55±0.41	91.02±0.36	98.20±0.25	99.30±0.20	86.70±0.37
VM-UNet	82.04±0.21	90.12±0.16	97.82±0.18	99.14±0.25	87.25±0.29
VM-UNetV2	82.75±0.35	90.72±0.43	98.10±0.29	99.25±0.23	91.10±0.38
MSCF-Net (Ours)	84.48±0.31	91.65±0.27	98.39±0.22	99.38±0.19	90.52±0.32
**ISIC 2017**					
**Model**	**mIoU** ↑	**DSC** ↑	**Acc** ↑	**Spe** ↑	**Sen** ↑
H-VMUNet	81.45±0.39	90.15±0.36	96.51±0.33	98.02±0.27	92.21±0.38
Mamba-UNet	78.12±0.38	88.14±0.35	95.89±0.39	97.68±0.32	86.85±0.36
VM-UNet	80.51±0.18	89.18±0.21	96.01±0.22	98.08±0.24	88.17±0.29
VM-UNetV2	80.76±0.34	89.79±0.41	96.42±0.30	98.49±0.35	88.82±0.31
MSCF-Net (Ours)	81.95±0.28	90.58±0.32	96.75±0.26	98.08±0.31	91.76±0.34
**ISIC 2018**					
**Model**	**mIoU** ↑	**DSC** ↑	**Acc** ↑	**Spe** ↑	**Sen** ↑
H-VMUNet	81.55±0.37	90.22±0.41	95.18±0.34	96.68±0.31	91.08±0.44
Mamba-UNet	79.35±0.39	89.12±0.33	94.62±0.35	96.48±0.26	88.67±0.37
VM-UNet	80.74±0.22	89.33±0.25	94.71±0.26	96.02±0.33	90.56±0.31
VM-UNetV2	81.38±0.32	89.92±0.38	95.03±0.36	96.21±0.35	92.14±0.40
MSCF-Net (Ours)	82.24±0.35	90.76±0.31	95.38±0.38	96.48±0.29	91.75±0.34

**Table 5:** Complexity and inference-speed comparison at an input size of 256×256. Params were counted from model parameters, GFLOPs were measured using THOP, and FPS was measured on an NVIDIA GeForce RTX 4090 with batch size 1. A downward arrow (↓) indicates that a lower value is better, and an upward arrow (↑) indicates that a higher value is better. The best value in each column is shown in bold.

Model	Params (M) ↓	GFLOPs (G) ↓	FPS ↑
Att-U-Net	24.55	7.86	78.96
DeepLabV3+	22.44	7.93	109.63
MA-Net	31.78	8.36	76.97
U-Net	7.70	41.71	**279.17**
UNet++	26.08	18.45	85.74
TransUNet	17.19	38.46	198.15
EGE-UNet	**1.04**	7.03	253.80
VM-UNet	27.43	4.11	32.52
VM-UNetV2	17.91	4.40	35.02
Mamba-UNet	15.48	4.60	51.56
H-VMUNet	8.97	**0.74**	8.45
MSCF-Net (Ours)	31.55	4.33	30.25

**Table 6:** Ablation results for MSCB and CLAF. Numbers denote DSC (%) on the three datasets. A checkmark indicates that the corresponding module is used, and an upward arrow (↑) indicates that a higher value is better. The best result in each column is shown in bold.

MSCB	CLAF	CVC-ClinicDB DSC (%) ↑	ISIC 2017 DSC (%) ↑	ISIC 2018 DSC (%) ↑
–	–	90.15	89.22	89.37
✓	–	90.88	89.95	90.12
–	✓	90.65	89.78	89.92
✓	✓	**91.72**	**90.62**	**90.82**

**Table 7:** Influence of MSCB placement and CLAF scope under the full model setting. Results are reported as DSC (%). An upward arrow (↑) indicates that a higher value is better. The best result in each column is shown in bold.

Configuration	CVC-ClinicDB DSC (%) ↑	ISIC 2017 DSC (%) ↑	ISIC 2018 DSC (%) ↑
Baseline (VM-UNet)	90.15	89.22	89.37
MSCB at Early Stage + CLAF at All Skips	90.72	89.85	89.98
MSCB at Bottleneck + CLAF at Single Skip	91.05	90.12	90.28
MSCB at Bottleneck + CLAF at All Skips (Ours)	**91.72**	**90.62**	**90.82**

**Table 8:** Complexity and performance of different module variants on CVC-ClinicDB. A downward arrow (↓) indicates that a lower value is better, and an upward arrow (↑) indicates that a higher value is better. The best result in each column is shown in bold.

Model	Params (M) ↓	GFLOPs (G) ↓	CVC-ClinicDB DSC (%) ↑
VM-UNet (Baseline)	27.43	4.11	90.15
+MSCB	31.23	4.29	90.88
+CLAF	27.75	4.15	90.65
MSCF-Net (Ours)	31.55	4.33	**91.72**

## Data Availability

The ISIC 2017 and ISIC 2018 datasets are publicly available from the ISIC Archive (https://www.isic-archive.com/, accessed on 28 June 2026). The CVC-ClinicDB dataset is publicly available from the CVC-ClinicDB benchmark repository (https://polyp.grand-challenge.org/CVCClinicDB/, accessed on 28 June 2026).
